# Alien Species and Human Health: Austrian Stakeholder Perspective on Challenges and Solutions

**DOI:** 10.3390/ijerph15112527

**Published:** 2018-11-12

**Authors:** Stefan Schindler, Wolfgang Rabitsch, Franz Essl, Peter Wallner, Kathrin Lemmerer, Swen Follak, Hans-Peter Hutter

**Affiliations:** 1 Environment Agency Austria, Spittelauer Lände 5, 1090 Vienna, Austria; wolfgang.rabitsch@umweltbundesamt.at (W.R.); franz.essl@umweltbundesamt.at (F.E.); 2 Division of Conservation Biology, Vegetation and Landscape Ecology, University of Vienna, Rennweg 14, 1030 Vienna, Austria; 3 Department of Environmental Health, Center for Public Health, Medical University of Vienna, Kinderspitalgasse 15, 1090 Vienna, Austria; peter.wallner4@gmail.com (P.W.); kathrin.lemmerer@meduniwien.ac.at (K.L.); hans-peter.hutter@meduniwien.ac.at (H.-P.H.); 4 Institute for Sustainable Plant Production, Austrian Agency for Health and Food Safety, Spargelfeldstraße 191, 1220 Vienna, Austria; swen.follak@ages.at

**Keywords:** *Aedes*, allergenic plants, *Ambrosia*, control, *Heracleum*, mosquitos, prevention, public health, questionnaire

## Abstract

No saturation in the introduction, acceleration of spread and the increasing impacts of alien species are a characteristic feature of the Anthropocene. Concomitantly, alien species affecting human health are supposed to increase, mainly due to increasing global trade and climate change. In this study, we assess challenges and solutions posed by such species to the public health sector in Austria over the next few decades. We did so using an online questionnaire circulated to 131 experts and stakeholders working on human health and biological invasions, supplemented by in-depth interviews with eleven selected experts. Results from the online survey and in-depth interviews largely support and complement each other. Experts and stakeholders suggest that (i) the allergenic *Ambrosia artemisiifolia* (common ragweed), the photodermatoxic *Heracleum mantegazzianum* (giant hogweed), and vectors of diseases such as *Aedes albopictus* (Asian tiger mosquito) are considered the alien species posing the most severe challenges; (ii) challenges are expected to increase in the next few decades and awareness in the public health sector is not sufficient; (iii) effective and efficient solutions are mainly related to prevention. Specific solutions include pathway management of introduction and spread by monitoring and controlling established populations of ragweed, hogweed and mosquitos.

## 1. Introduction

Alien species may cause substantial negative impacts on biodiversity and human well-being [1,2,3,4]. They also present a challenge for environmental policy [5,6]. Most likely, related challenges will increase in the future due to increasing economic activities, trade and transport. This will result in many new introductions of alien species [7], and the pool of potentially available alien species will increase further [8]. Concomitantly, other drivers of global environmental change such as climate change [9,10], habitat deterioration [11] and eutrophication [12] will likely foster biological invasions.

Some alien species including endoparasites—such as fleas, ticks and mites, cause baylisascariasis, strongyloidiasis, coenurosis, and ectoparasites. These species may present new challenges for human health and public health systems [13]. Alien species affect human health in several ways; they cause diseases or infections, expose humans to wounds from bites or stings, biotoxins, allergens or toxicants [14,15]. They might also lead to indirect effects due to changes induced in ecosystems [14]. In Europe, alien species causing health impacts mainly include allergenic plants and mosquitos that can function as vectors of disease [16]. Alien allergenic plants such as *Ambrosia artemisiifolia* (common ragweed) cause a prolonged pollen season, which relates to suffering for sensitive individuals, and high socioeconomic costs due to sickness leaves and treatment [17]. Alien vectors of disease are responsible for transmission of pathogens that until recently have not been present, or they contribute to a higher prevalence of existing vector-borne diseases [18,19]. Further health-relevant alien species include ticks, algae, jellyfish, wasps and moths [16,20,21].

Invasion science is still dominated by natural sciences, while multidisciplinary approaches are rare [22]. Bayliss et al. [21] screened over 15,700 articles, yet only sixteen of them provided evidence for changes in the occurrence, frequency or severity of human health impacts resulting from exposure to alien species in Europe. This was due to the fact that many of them assessed either species population trends or health impacts, but hardly both. This knowledge gap hinders an appropriate understanding of the processes that cause biological invasions, and is specifically detrimental for identifying and implementing management solutions. In particular, the relationships between spread and abundance of health-relevant species, and their impact on human health and management are insufficiently investigated [21].

Here, we assess the challenges posed by alien species relevant to human health and human health systems for the decades to come. We accomplish this by consulting Austrian experts and stakeholders working on human health and biological invasions using an online questionnaire and in-depth interviews. Austria has been chosen because it is particularly prone to the introduction of alien species as a result of the accelerated import of goods and commodities from overseas and from other European countries. Due to its location on the crossroads of the continent, intensive transport of goods and commodities takes place, and a naturally diverse and heterogeneous landscape mosaic provides suitable environments for an increasing number of alien species [23]. Questions asked related to the importance of challenges by, and solutions to alien species’ impacts on human health. Specifically, we assessed (i) which species are considered severe challenges for human health; (ii) how human health and public health systems are currently affected and might be affected in the future; and (iii) which measures should be taken to get prepared for these challenges and to overcome them.

## 2. Methods

In this study, we applied expert assessments that are preferred knowledge synthesis methods for explorative questions, particularly under constraints in terms of funding and time [24,25]. The expert assessments combined two approaches: First, an online questionnaire with eight closed questions, which was circulated to 131 experts and stakeholders working in Austria. Online questionnaires are widely used for obtaining knowledge from a broader set of stakeholders [26,27,28], and their application has strongly increased in most fields of science during the last years. Second, we used in-depth interviews with open questions following guidelines for a structured interview. In-depth interviews are particularly suitable for obtaining more specific information from a rather small number of key experts on an issue of limited scope [29]. The combination and complementarity of these two methods is beneficial because it guarantees a comprehensive overview on expert and stakeholder opinions. It also provides specific insights that are not yet mainstream within the community of experts.

### 2.1. Online Questionnaire

In the online questionnaire we asked the following closed questions and provided predefined response options, as seen in Supplementary File S1. These were both provided originally in German language: (i) Are alien species that affect human health a topic in your working responsibilities?; (ii) Which health-relevant alien species do you consider to be the most important?; (iii) How important do you consider the following specific health effects of alien species?; (iv) How important do you consider the following general effects of alien species on the public health system?; (v) Have you been discussing measures against health-related effects of alien species in your institution?; (vi) Have you been implementing measures against health-related effects of alien species in your institution?; (vii) Have you been facing any obstacles or problems with their implementation?; (viii) Which measures can you recommend for future implementation?

We sent the questionnaire to independent experts working in different relevant sectors such as health, environment, research, education, and public administration. Thus, experts covered a wide spectrum of different skills, experience and knowledge on the challenges posed by alien species for human health. We used the software Qualtrics (Qualtrics LLC, Provo, UT, USA; www.qualtrics.com) to compile and distribute the online questionnaire, and analyzed the responses descriptively. For questions (iii) and (iv) we used a four step Likert scale [30] with the options being very important, important, less important, not important.

### 2.2. In-Depth Interviews

Structured in-depth interviews were conducted with selected renowned medical experts (*n* = 11) with large experience in the fields of public health, microbiology, and certain medical disciplines (such as allergology). They covered a broad field of experience and knowledge. Expert selection was based on our long-lasting work experience in the field of environmental and public health. We believe that we have covered most of the national experts in the field, as the number of Austrian experts in public health is rather limited. We developed an interview guide with open questions, which focused mainly on the following issues: Which health-relevant alien species do you consider the most important? Which health effects of alien species do you consider the most important? Do you think that negative effects of alien species on human health will occur more frequently in the future? Is the health system prepared for this? Is awareness of the problem of alien species and health sufficiently high (among physicians, politicians, and the wider public)? What measures can you recommend for future implementation?

Each in-depth interview lasted for approximately one hour. Interviews were conducted by medical students, and experts both from the field of environmental and public health.

## 3. Results

### 3.1. Online Questionnaire

The survey was available online from 23 May 2016 to 7 September 2016. We invited 131 Austrian experts to participate in the survey, of which 53 responded (40% return rate). However, not all of the 53 responded to each question of the survey. Eleven of the respondents (21%) specified health-relevant alien species as important responsibility in their daily work.

Regarding the question on their fields of action, 51 respondents provided 99 answers. Fifty-nine (59%) of respondents were active in the environmental sector, 47% in public administration, 35% in research, 25% in medicine, 24% in education, and 4% in other sectors.

Forty-nine respondents provided 149 answers to the question regarding most relevant alien species groups. Of these, 88% assumed allergenic plants to be relevant, 55% opted for “other plants”, 51% for invertebrate disease vectors, 45% for pathogens, 27% for vertebrate disease vectors, and 27% for other species groups than those mentioned until now.

**Figure 1:**
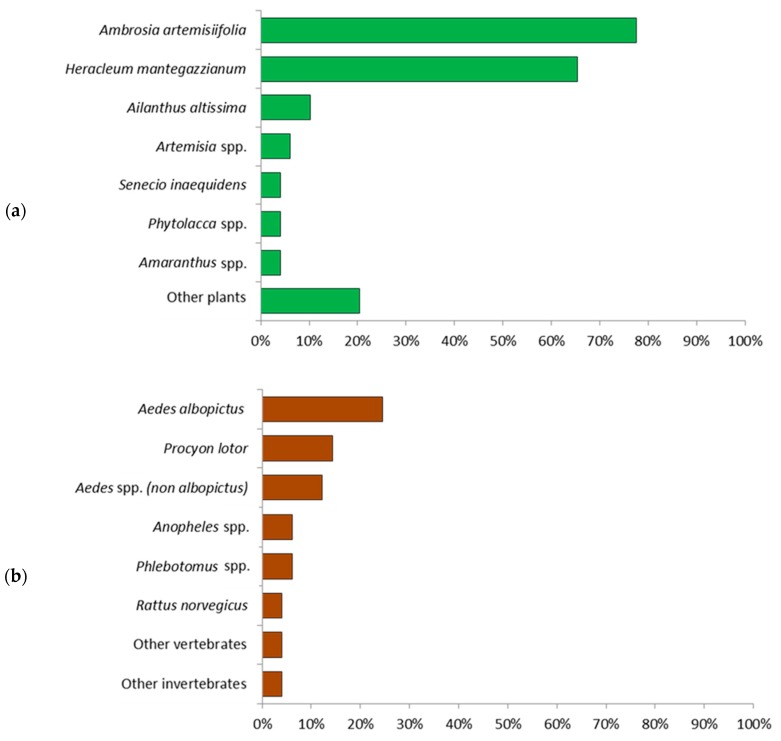
Alien plant (**a**) and animal (**b**) species that were considered health-relevant (*n* = 49 respondents) (multiple answers were allowed).

When asked for particularly relevant alien plant species, 78% of 49 respondents mentioned *Ambrosia artemisiifolia* and 65% mentioned *Heracleum mantegazzianum*. Other plant species were mentioned only by few participants, as seen in Figure 1a. Regarding particularly relevant alien animal species, 24% of 49 respondents mentioned *Aedes albopictus*, 14% *Procyon lotor* (racoon) and 12% *Aedes* spp. other than *Ae. Albopictus*, as seen in Figure 1b.

**Figure 2:**
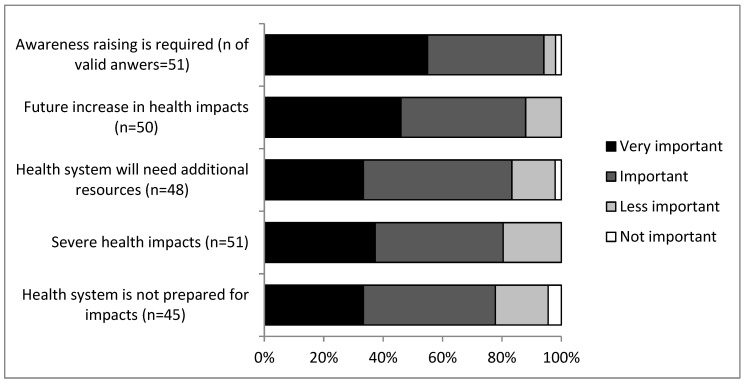
Level of importance of challenges caused by alien species to the Austrian health system as reported by stakeholders (n values refer to valid answers of stakeholders that chose one of the four Likert scale categories).

Figure 2 represents a summary of collected data on the level of importance of challenges caused by alien species. The data shows that a total of 94% of the respondents stated that raising the awareness on the health impacts of alien species is “very important” or “important”, 88% stated that a future increase in health impacts is considered likely, 83% stated that the health system will need additional resources, 80% stated that the health impacts of these species will be severe, and 78% stated that the health system is not prepared for expected impacts.

**Figure 3:**
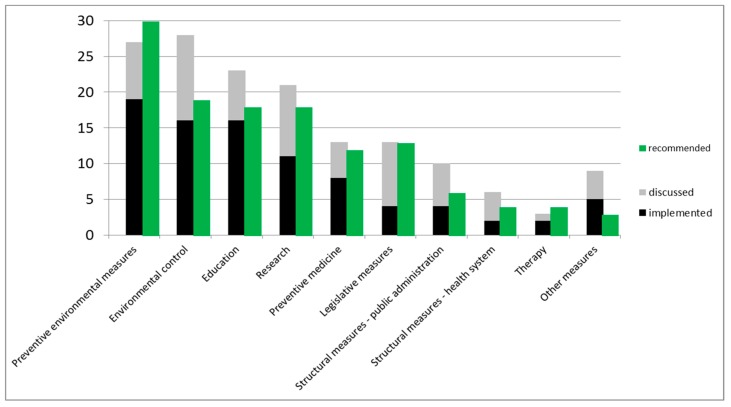
Which kind of measures were discussed or implemented in your institution? Which kind of measures could you recommend? (*n* = 51–52 respondents).

Three complementary questions were asked about measures against health-relevant alien species, i.e., whether such questions had already been discussed, implemented, or can be recommended in the working environment of the interviewees. The rationale behind this is that any kind of measures are usually discussed in institutions before being implemented. Their implementation can be recommended by experts participating in the online survey with or without previous discussions or implementation. These questions were answered by 51–52 respondents. Preventive environmental measures were often implemented (37%), discussed (52%) and recommended (58%) by respondents or their institutions, as shown in Figure 3. In addition, measures related to environmental control were often mentioned (31% implemented, 54% discussed, and 37% recommended). Measures relating to education were implemented by 31%, discussed by 44%, and recommended by 35% of the respondents. Research on health-relevant alien species was implemented by 22%, discussed by 40%, and recommended by 35% of the respondents. Legislative measures, structural measures in public administration and the public health system, and therapies were implemented, discussed and recommended to a lesser extent, as shown in Figure 3. These data also show that all types of measures were more frequently recommended than currently implemented with particularly large discrepancies (i.e., “implementation gap”) for preventive environmental measures and legislative measures.

For the open question “Which measures you can recommend for implementation in the future”, we received 104 responses covering all aspects specified in Figure 3 and Supplementary File S2.

### 3.2. In Depth Interviews

The main health-relevant alien species from the perspective of the interviewed experts were common ragweed, giant hogweed, *Aedes aegypti,* and *A. albopictus*. In addition, other mentioned species included the West Nile virus, parasites such as leishmania, *Impatiens glandulifera* (Indian balsam), *Toxicodendron radicans* (poison ivy), *Ficus benjamina* (weeping fig), antibiotic resistant germs, hornets, and spiders. Allergies and infections from viruses were indicated to have the most important health effects.

All experts stated that negative health effects of alien species will occur more frequently in the future (likely to virtually certain). Reasons mentioned were climate change, globalization and uncritical introduction, and the use of alien species. Opinions were divided on the health systems state of preparation. From the experts opinion, problem awareness is not sufficient among citizens, politicians, and the majority of physicians. However, some experts also stated that problem awareness is unbalanced, especially that the current risks of emerging infectious diseases in Austria were overestimated by the public and media. 

The specific measures recommended by the experts were diverse and included (i) measures of pathway control such as trade and transport restrictions; (ii) surveillance and intensification of border controls and monitoring (e.g., pollen and mosquitos); including development of watchlists for alien plants, rapid adoptions of the Protection against Infection Act and the obligation to notify of emerging diseases, and the enforcement of the obligation to notify; (iii) measures of management and control such as intensification of ragweed management; (iv) raising awareness of citizens and training of physicians; (v) measures related to clarification of responsibilities in sectoral public administration and improved collaboration among professionals from different sectors (environment, medicine, and agriculture); and (vi) the general advice to implement already existing recommendations.

## 4. Discussion

Experts and stakeholders who responded to the online questionnaire clearly identified the most important species groups and species, i.e., common ragweed, giant hogweed, and the Asian tiger mosquito. Also, in the in-depth interviews allergies and infections from viruses were mentioned as the most important health effects. This is in concordance with results from a recent literature review at European scale that identified allergenic plants and disease vectors as the most relevant alien species groups with human health impacts [16]. Indeed, public health impacts of the aforementioned alien species are known and have been quantified in Austria in some detail [17,31].

Data in this paper have led to stronger concerns about the future than about the current situation. This indicates that the topic is still considered a secondary challenge at the moment, but with increasing relevance for the future. The European literature on alien species relevant to human health suggests that the magnitude of the impacts is increasing. However, often-direct evidence is lacking, and this trend is inferred indirectly from data on increasing ranges and abundance of the species [16,21]. In Austria, increasing impacts and new threats are partly mirrored by available data. In total, 25–30% of individuals allergic to pollen react to ragweed in Eastern Austria, where the species has its strongholds [32]. An evaluation of allergy test results in Eastern Austria showed a continuous increase of the sensitization rate to ragweed pollen from 8.5% in 1997 to 17.5% in 2007. The sensitization rate is predicted to further increase in the future [17,31]. Alien mosquito species that are competent disease vectors have been detected recently in Austria [33]. *Ochlerotatus japonicus* was first detected in 2011 and the Asian tiger mosquito in 2012 [34], both were introduced by trade. The Mediterranean mosquito species *Anopheles hyrcanus* and *Culiseta longiareolata* probably benefitted from climate change; *Anopheles hyrcanus* is meanwhile established in the Federal States of Vienna, Lower Austria and Burgenland [33,35,36], *Culiseta longiareolata* was detected in Styria [37] and Lower Austria [38]. Additionally, new mosquito-borne pathogens have been recently detected, particularly filaroid helminths including *Dirofilaria repens* and West Nile Virus, and some of these cases were discussed to be autochthonous and not imported [39,40,41,42]. Between 2009 and September 2018, 41 cases of autochthonous human West Nile virus infections were recorded in Austria [42]. Data on case numbers of individuals with skin lesions caused by giant hogweed per year are not available for Austria. Therefore, we recommend establishing a monitoring system as a basis for targeted risk assessment [43].

Seemingly, the public health system is not well prepared for increasing impacts of alien species. This was stated by the respondents of the online survey and by the public health experts asked in the in-depth interviews. It is indeed expected that the burden of health-relevant alien species will increase. Successful alien species are characterized by traits that facilitate spread and survival in new areas [44]. Currently, there is no sign of saturation of increasing populations of alien species in Europe or elsewhere [7,45]. Large species pools of potentially alien species are still to be reached, and new and increasing trade routes and emerging pathways will cause increasing introductions of emerging alien species [8,46]. Finally, climate change will facilitate establishment and further spread of alien species in general [47], but also of health-relevant alien species such as *Ambrosia artemisiifolia* [48,49] and *Aedes aegypti* [50] in particular. In a recent review, Schindler et al. [51] concluded that climate change will significantly facilitate the establishment, spread, and impact of health-relevant alien species in Europe, while introduction of such species will only be facilitated moderately.

Given the expected increase in the number of health-relevant alien species, their distribution ranges and their impacts, surveillance must be considered as a particularly important measure to be undertaken. Thus, existing efforts of mosquito monitoring in Austria [33,52] should be intensified, and extended to surveillance activities for other relevant species groups. Preventative environmental measures and environmental control were recommended by the respondents of the online survey as well as by environmental and public health experts. Indeed, it should be most efficient to counteract the species before they arrive and become established. Thus, prevention of introduction and establishment by pathway control and rapid response measures is considered the most efficient strategy against alien species [53]. Further environmental measures to prevent health impacts should include limiting further spread of ragweed by consequent surveillance, and the application of a range of physical and chemical control options [54]. Such dedicated management is predicted to result in substantial socio-economic benefits, over-compensating by far the costs incurred by management [17]. In addition, raising awareness among the general public and particularly relevant groups of citizens has been considered as an important measure of environmental prevention as shown in Supplementary File S2. In-depth interviews with experts in this study confirmed the claims of Richter et al. [17] that it is crucial and urgent to reduce further geographical expansion of ragweed, because increases in the amount of pollen and allergenic potency due to climate change have to be taken into consideration. In addition, control of Giant Hogweed is well investigated and rather effective to be implemented [55]. Alien pathogens and disease vectors often interact in complex ways, with several species being involved [19]. A single alien vector might transmit several native or alien pathogens that occur in several native or alien reservoir hosts, however, for many recently emerging human diseases the pathogens were introduced by humans functioning as reservoir hosts [18]. The complexities of such host-pathogen systems cause substantial uncertainties (in analogy to wildlife pathogens) [56].

Given the number of responses, the return rate, and the diversity of sectors where respondents are active, we consider that the online survey and the in-depth interviews were representative for the current knowledge of alien species with human health impacts and their relevance for the public health system in Austria. In general, the results (e.g., awareness raising and communication within the administration) of the in-depth interviews are not surprising. However, the output confirms specific needs, which should be communicated to decision makers more vigorously. In this respect, awareness for alien species other than common ragweed and giant hogweed should be increased, for instance by awareness raising campaigns and trainings for particularly relevant professions, such as physicians. Furthermore, raising awareness among the general public was considered highly relevant.

## 5. Conclusions and Recommendations

The results of this study also reveal that collaborations and cooperation among environmental and medical researchers are important for exchanging and connecting meaningful knowledge across scientific domains [57]. Despite the emerging field of environmental health, collaboration between environmental and medical researchers is still limited. Hardly any articles investigate simultaneously the environmental and medical aspects of biological invasions [20,22]. In particular, strong evidence is often lacking on how health impacts depend on increasing spread and densities of health-relevant alien species [21]. Thus, we recommend that collaboration and cooperation among environmental and medical researchers should be enhanced, e.g., by establishing a joint communication platform or supporting interdisciplinary project proposals within Austrian science funding schemes. Finally, we argue that the insights gained in the Austrian context will be informative for other countries facing similar challenges to human health posed by alien species.

## Supplementary Materials

Supplementary material published online at http://www.mdpi.com/1660-4601/15/11/2527/s1. Alongside the manuscript includes File S1: Questions and predefined response options of the online questionnaire, File S2: Recommendations for future implementation.
