# Supplementary material for: Alien Species and Human Health: Austrian Stakeholder Perspective on Challenges and Solutions
Source: Int J Environ Res Public Health. 2018 Nov 12;15(11):2527. doi: 10.3390/ijerph15112527 (PMC6266649; doi:10.3390/ijerph15112527)
Supplement: Supplementary file 1 [file ijerph-15-02527-s001.pdf]

Alien species and human health: Austrian stakeholder perspective on challenges and solutions

Schindler S. et al.

Supplementary Material 1

Questions and predefined response options of the online questionnaire

Question 1: Are alien species that affect human health a topic in your working responsibilities?

- ❍ no
- ❍ yes
- ❍ n.a.

Question 2: Which health-relevant alien species do you consider as the most important (multiple answers possible)?

- ❑ allergenic plants. Which? ____________________
- ❑ other plants. Which? ____________________
- ❑ pathogens. Which? ____________________
- ❑ invertebrate disease vectors. Which? ____________________
- ❑ other invertebrates. Which? ____________________
- ❑ vertebrate disease vectors. Which? ____________________
- ❑ other vertebrates. Which? ____________________
- ❑ other organisms. Which? ____________________
- ❑ n.a.

Question 3: How important do you consider the following specific health effects of alien species?

	Very important	Important	Less important	Not important	n.a.
Transmission of diseases and infections	❍	❍	❍	❍	❍
Allergies	❍	❍	❍	❍	❍
Toxic reactions	❍	❍	❍	❍	❍
Other impacts. Which? (1)	❍	❍	❍	❍	❍
Other impacts. Which? (2)	❍	❍	❍	❍	❍
Other impacts. Which? (3)	❍	❍	❍	❍	❍

Question 2.4: How important do you consider the following general effects of alien species on the public health system?

	Very important	Important	Less important	Not important	n.a.
Severe health impacts	❍	❍	❍	❍	❍
Health impacts are occurring more and more frequently	❍	❍	❍	❍	❍
Future increase in health impacts	❍	❍	❍	❍	❍
Impacts increasingly pose Challenger for the health sytysem	❍	❍	❍	❍	❍
Health system will need additional resources	❍	❍	❍	❍	❍
Health system is not prepared for impacts	❍	❍	❍	❍	❍
Awareness raising is required	❍	❍	❍	❍	❍
Other impacts. Which? (1)	❍	❍	❍	❍	❍
Other impacts. Which? (2)	❍	❍	❍	❍	❍
Other impacts. Which? (3)	❍	❍	❍	❍	❍

Question 2.5: Have you been discussing measures against health-related alien species in your institution (multiple answers possible)?

- ❑ No, none.
- ❑ Yes, preventive environmental measures. For instance: ____________________
- ❑ yes, environmental control. For instance: ____________________
- ❑ yes, preventive medicine. For instance: ____________________
- ❑ yes, therapies. For instance: ____________________
- ❑ yes, education. For instance: ____________________
- ❑ yes, research. For instance: ____________________
- ❑ yes, legislative measures. For instance: ____________________
- ❑ yes, structural measures in the health system. For instance: ____________________
- ❑ yes, structural measures in public administration. For instance: ____________________
- ❑ yes, other measures. For instance: ____________________
- ❑ n.a.

Question 6: Have you been implementing measures against health-related alien species in your institution (multiple answers possible)?

- ❑ No, none.
- ❑ Yes, preventive environmental measures. For instance: ____________________
- ❑ yes, environmental control. For instance: ____________________
- ❑ yes, preventive medicine. For instance: ____________________
- ❑ yes, therapies. For instance: ____________________
- ❑ yes, education. For instance: ____________________
- ❑ yes, research. For instance: ____________________
- ❑ yes, legislative measures. For instance: ____________________
- ❑ yes, structural measures in the health system. For instance: ____________________
- ❑ yes, structural measures in public administration. For instance: ____________________
- ❑ yes, other measures. For instance: ____________________
- ❑ n.a.

Question 7: Have you been facing any obstacles or problems with their implementation?

- ❍ n.a. (no measures have been implemented)
- ❍ no, we did not face any obstacles or problems
- ❍ yes, we faced obstacles or problems during implementation. Which ? ______________________
- ❍ n.a.

Question 8: Which measures can you recommend for future implementation (multiple answers possible)?

- ❑ No, none.
- ❑ Yes, preventive environmental measures. For instance: _____________________
- ❑ yes, environmental control. For instance: _____________________
- ❑ yes, preventive medicine. For instance: _____________________
- ❑ yes, therapies. For instance: _____________________
- ❑ yes, education. For instance: _____________________
- ❑ yes, research. For instance: _____________________
- ❑ yes, legislative measures. For instance: _____________________
- ❑ yes, structural measures in the health system. For instance: _____________________
- ❑ yes, structural measures in public administration. For instance: _____________________
- ❑ yes, other measures. For instance: _____________________
- ❑ n.a.

Question sector: In which sectors do you work (multiple answers possible)?

- ❑ health sector
- ❑ environment sector
- ❑ research
- ❑ education
- ❑ administration
- ❑ public administration
- ❑ other. Which? ____________________
- ❑ n.a.

Supplementary Material 2

Recommendations for future implementation

Category	Recommendation
**Environmental prevention**	
	Appropriate biotope management - also outside protected areas
	Appropriate biotope management to prevent further spread monitoring
**3 x**	Awareness raising
	Central reporting center for health relevant neobiota
	Combat emerging (potentially) invasive alien species from the beginning
	Control of imports
	Creating awareness also in schools
	Detecting and removing allergenic neophyta early, when populations are still relatively small
	Education of the population, control of seed (ragweed) if possible
	Expansion of education, monitoring, mapping and early warning systems
	Implementation of a national plan for the early detection and control of health-related neobiota
	Increased climate protection
	Increased training and information in municipalities
	Information for garden owners
	Information, capacity building, training (including in forestry, agriculture), monitoring, networking of public authorities
	Information, data collection, data interpretation
	Monitoring of hibernating mosquitos - possibly control measures
	Monitoring of neobiota
	More funding for vector surveillance
	Prevention of spread (e.g. ban on birdseed contaminated with ragweed seeds)
	Prevention of the further spread of health relevant neobiota, definition of introduction pathways, early warning system, action plans for emerging alien species and species already in neighbouring countries, information campaigns

	Repeated detailed information of the population about dangers and handling of (potentially) invasive alien species, e.g. via public media such as TV, community newspaper, brochures, bulk mail, etc.
	Reporting centers for the occurrence of neobiota at federal level
	Vector control
**Environmental control**	
	Action days to combat invasive neophytes involving municipalities
	Active control: Extraction, excavation, mowing, use of herbicides. Observing and, if necessary, repeating the action
	Adapted timing of mowing in all regions affected by ragweed
	Appropriate control (e.g. ragweed control: to root out with the roots)
	Consistent implementation of existing management plans
	Control of populations
	Cutting out of giant hogweed; ripping of ragweed; prevention of spread of contaminated earth material
	Eradication of *Ambrosia artemisiifolia* along traffic routes in a targeted and timely manner
	Information and guidance for municipalities and garden owners
	Legally anchored management of problematic species
	Mosquito control (standing waters)
	Neophyte aftercare and precaution at major construction sites
	Neophyte control applying various techniques (scouring, mowing, ringing, ...)
	Ragweed control
	Reduction of neophytes
	Removal of invasive species, where appropriate - preliminary clarification of costs, benefits, efficiency and relevance => health risk, dispersal potential, sustainability of the measures
	Removal of knotweed
	Removal of relevant plants or breeding grounds of relevant animal species
	Stronger involvement of the population
**Education**	
	Awareness raising about alien species, particularly in relation to infectious diseases
	Awareness raising among citizens
	Force identification of health relevant alien species by the general public
	Further training if required by the responsible authorities
	Information events in schools, communities, nature parks

	Information of affected professional groups
	Internet information of citizens and target groups
	Regular training of practitioners
	Regular training sessions for the public health service
	Regular updates on status and trends of relevant species would be desirable
	Scientific networking
	Special training on ragweed for the concerned road service authorities and other authorities of the other federal states
	Stronger technical preparation of this topic, more events, training
	Training of employees of municipalities, nature guards, farmers, gardeners
	Workshops for specific groups, excursions
**Research**	
	Development of monitoring systems, prognosis models, studies to characterize vulnerable groups of citizens
	Early detection through taxonomic expertise and molecular methods
	Efficiency of control measures
	Further master theses and other studies
	Implementation of various methods (without use of pesticides)
	Increased research budget for applied research
	Optimization of control measures, improved scientific bases of biotope management
	Projects on ecology, management and risk analysis of neobiota
	Research on distribution and spread of relevant neophytes in Tyrol in the form of dissertations, master and bachelor thesis
	Status survey and interpretation
	Symptom forecasting (e.g. for allergic persons)
	Targeted calls in funding programs
	Vector surveillance should be of the highest scientific standard
**Preventive medicine**	
	Appropriate training and awareness raising
	Awareness raising campaigns, especially on Ambrosia, training of medical professionals, pharmacists
	Diagnostic measures (preparation for various pathogens to arrive)
	Improvement of modelling
	Information

	Information, capacity building in public health systems, monitoring systems, development of forecasting models, networking of public authorities
	More funding for vector-associated pathogens
	Mosquito repellent
	Risk assessment
	Vaccinations
**Legislative measures**	
	Anchoring of neophytes in nature conservation laws of federal states or nationwide legislation
	Ban on the release of harmful (and other) neobiota: regulations for construction companies
	Better anchoring of legal measures to health-relevant neobiota
	Extension of mandatory reporting
	Implementation of the polluter pays principle
	Legal definitions on dealing (e.g. control, disposal) with particularly invasive species
	Mandatory reporting of ragweed and regulations for mandatory elimination
	Monitoring and reporting obligations of competent institutions (road traffic administration, water traffic administration, federal railway services ...)
	Obligatory control of large populations
	Reporting obligations
	Responsibilities for control measures need to be clarified
**Structural measures - public administration**	
	Collection of reports of occurrences
	Establishment of a contact point for newly introduced species
	Information for municipality workers
	Neobiota have been considered in the job descriptions of public administration of the Federal State Tyrol
**Structural measures - health system**	
	Education of affected occupational groups
	Increase attractiveness of public health work for medical doctors
**Therapy**	
	Extended approvals of drugs
	Therapies with respect to respiratory illnesses, impairments due to aggravation of allergens (ragweed); securing human resources

	Vaccinations (e.g. related to allergies)
**Other measures**	
	Awareness raising among the general public and the public administration
